# MiR-373 drives the epithelial-to-mesenchymal transition and metastasis via the miR-373-TXNIP-HIF1α-TWIST signaling axis in breast cancer

**DOI:** 10.18632/oncotarget.4702

**Published:** 2015-07-01

**Authors:** De Chen, Bian-Li Dang, Jin-zhou Huang, Min Chen, Di Wu, Man-Li Xu, Rong Li, Guang-Rong Yan

**Affiliations:** ^1^ Biomedicine Research Center and Department of Surgery, The Third Affiliated Hospital of Guangzhou Medicine University, Guangzhou, China; ^2^ Institutes of Life and Health Engineering, Jinan University, Guangzhou, China; ^3^ Cancer Center, Nanfang Hospital, Southern Medical University, Guangzhou, China; ^4^ Key Laboratory for Major Obstetric Diseases of Guangdong Province and Key Laboratory of Reproduction and Genetics of Guangdong Higher Education Institutes, The Third Affiliated Hospital of Guangzhou Medicine University, Guangzhou, China

**Keywords:** miR-373, EMT, TXNIP, ROS, TWIST

## Abstract

Our previous proteomics study revealed that thioredoxin-interacting protein (TXNIP) was down-regulated by miR-373. However, little is known of the mechanism by which miR-373 decreases TXNIP to stimulate metastasis. In this study, we show that miR-373 promotes the epithelial-to-mesenchymal transition (EMT) in breast cancer. MiR-373 suppresses TXNIP by binding to the 3′UTR of TXNIP, which in turn, induces cancer cell EMT and metastasis. TXNIP co-expression, but not the TXNIP-3′UTR, reverses the enhancement of EMT, migration, invasion and metastasis induced by miR-373. MiR-373 stimulates EMT, migration and invasion through TXNIP-dependent reactive oxygen species (ROS) reduction. Mechanistically, miR-373 up-regulates and activates the HIF1α-TWIST signaling axis via the TXNIP pathway. Consequently, TWIST induces miR-373 expression by binding to the promoter of the miR-371-373 cluster. Clinically, miR-373 is negatively associated with TXNIP and positively associated with HIF1α and TWIST, and activation of the miR-373-TXNIP-HIF1α-TWIST signaling axis is correlated with a worse outcome in patients with breast cancer. This signaling axis may be an independent prognostic factor for patients with breast cancer.

## INTRODUCTION

Breast cancer is the most frequent malignancy occurring in women and the second leading cause of cancer mortality in women worldwide. Approximately 90% of breast cancer-associated deaths result from the distant metastasis of primary tumors [1]. Metastasis is a complex process comprising multiple sequential steps and the regulation of multiple factors. Local invasion is considered an initial, indispensable step during the process of distant metastasis from primary tumors. The epithelial-mesenchymal transition (EMT) is a crucial step in the cancer metastasis process [2, 3].

MicroRNAs (miRNAs) are an abundant class of small non-coding RNAs consisting of 20-24 nucleotides that suppress gene expression at the post-transcriptional level by blocking mRNA translation or degrading target mRNAs [4, 5]. MiRNAs are also involved in both the promotion and suppression of cancer metastasis [6–8].

MiRNA-373 was first identified as a potential oncogene that promotes cellular transformation in testicular germ-cell tumors, in part through inhibiting the tumor suppressor, LATS2 [9]. Subsequently, miR-373 was shown to induce cancer invasion and metastasis, in part by targeting CD44 [10]. However, as demonstrated by Huang et al., miR-373 and miR-520c promote migration and invasion only in part by limiting CD44 expression in breast cancer [10], indicating that other targets may correlate with the regulation of miR-373 for invasion and metastasis. Our previous proteomics study found that miR-373 stimulated cancer cell migration and invasion, but not cell proliferation and cell cycle. Furthermore, many potential target genes, including TXNIP, were significantly down-regulated by miR-373 [11]. However, little is known about the mechanism by which miR-373 decreases TXNIP to stimulate cancer metastasis.

In this study, we demonstrate that miR-373 stimulates breast cancer cell EMT and metastasis by directly inhibiting TXNIP. MiR-373 increased HIF1α and TWIST via the TXNIP pathway to stimulate EMT. Subsequently, TWIST bound to the promoter of the miR-371-373 cluster to induce miR-373 expression. The activation of the miR-373-TXNIP-ROS-HIF1α-TWIST signaling axis is associated with a worse outcome in patients with breast cancer.

## RESULTS

### MiR-373 stimulates breast cancer cell EMT

**Figure 1:**
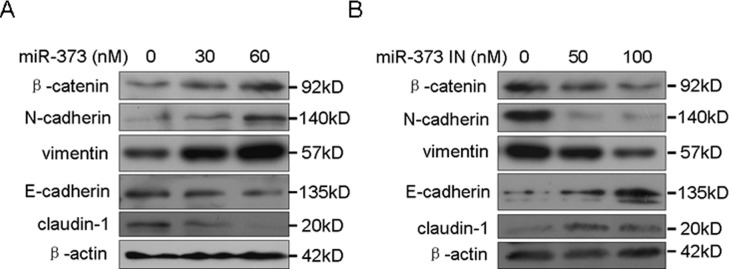
MiR-373 promotes cancer cell EMT **A.** MCF-7 cells were transfected with the miR-373 precursor at the indicated concentration, and the EMT markers were then detected by Western blotting. **B.** MDA-MB-231 cells were transfected with the miR-373 inhibitor (miR-373 IN) at the indicated concentration, and the EMT markers were then detected by Western blotting.

Our previous study showed that miR-373 promotes breast cancer metastasis [11]. EMT is a crucial step during cancer metastasis [2]. Therefore, the effects of miR-373 on EMT were further investigated. We demonstrated that miR-373 increased the levels of the mesenchymal markers, β-catenin, N-cadherin and vimentin, and decreased the level of the epithelial markers, E-cadherin and claudin, in a dose-dependent manner (Figure 1A), whereas inhibition of miR-373 had the opposite effect (Figure 1B). These observations indicate that miR-373 promotes cancer metastasis by inducing EMT.

### MiR-373-induced EMT and metastasis occurs primarily through inhibiting TXNIP

Our previous proteomics analysis demonstrated that miR-373 decreases TXNIP [11]. Here, we further tested whether TXNIP is the main target responsible for miR-373-induced EMT. As shown in Supplementary Figure S1A, TXNIP was reduced in the metastatic MDA-MB-231 and MDA-MB-435s cells compared to the non-metastatic MCF-7 cells and was negatively correlated with the level of miR-373 (Supplementary Figure S1B). TXNIP was also down-regulated by miR-373 and was increased when miR-373 was inhibited (Supplementary Figure S1C, S1D).

The luciferase reporter constructs containing the TXNIP 3′UTR were transfected into MCF-7-373 cells stably expressing miR-373. MiR-373 over-expression decreased the luciferase activity (Supplementary Figure S1E), whereas the luciferase activity was not changed when the 3′UTR sequences in the complementary sites for the seed region of miR-373 were mutated (Supplementary Figure S1E). Furthermore, when the constructs were co-transfected into cells together with an anti-miR-373 inhibitor, the anti-miR-373 inhibitor significantly increased the luciferase activity (Supplementary Figure S1F). The anti-miR-373 inhibitor did not change the luciferase activity of TXNIP when the 3′UTR sequences in the complementary site for the seed region of miR-373 were mutated (Supplementary Figure S1F). Taken together, our data demonstrate that TXNIP is a direct target of miR-373.

**Figure 2:**
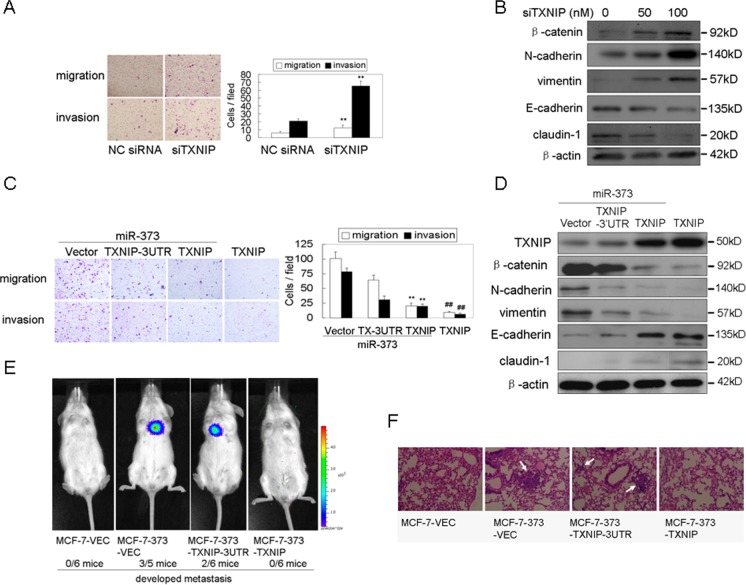
MiR-373 induces EMT, migration, invasion and metastasis by directly inhibiting TXNIP **A.**, **B.** MCF-7 cells were transfected with anti-TXNIP siRNAs at the indicated concentration, and then the migration and invasion **A.** and EMT markers **B.** were analyzed as indicated (*n* = 3). **C.**, **D.** MCF-7 cells were co-transfected with the indicated plasmids and 30 nM miR-373 precursor, and then migration and invasion **C.** and EMT markers **D.** were analyzed as indicated (*n* = 3). **E.** NOD-SCID mice were transplanted with the indicated Luc-labeled cells (4×10^6^ cells/mouse) via tail vein injection (*n* = 6), and these mice were then visualized ten weeks post-transplantation using the IVIS 200 Imaging System. **F.** Histological analysis of the pulmonary metastases in the animal model as described in Figure 2E.

The silencing of TXNIP induced EMT and promoted migration and invasion in breast cancer cells (Figure 2A, 2B), similar to the effects of miR-373 over-expression. Furthermore, miR-373 mimics were co-transduced into non-metastatic MCF-7 cells together with the TXNIP vector or TXNIP-3′UTR vector. The TXNIP level was increased in the TXNIP-transfected cells because the miR-373 had no effect on the expression of TXNIP due to the absence of miR-373 binding sites, whereas the TXNIP level was weakly up-regulated in the TXNIP-3′UTR-transfected cells because the expression of TXNIP 3′UTR was inhibited by the co-transfected miR-373 (Figure 2D). Co-transfection of TXNIP with miR-373 mimics, but not the TXNIP 3′UTR, reversed the enhancement of EMT, migration and invasion induced by miR-373 (Figure 2C, 2D).

Furthermore, the roles of TXNIP in miR-373-induced metastasis were measured *in vivo*. Luciferase-tagged MCF-7 cells stably expressing miR-373 with TXNIP or TXNIP-3′UTR were transplanted into NOD-SCID mice via tail-vein injection. Metastatic nodules developed in the lungs or skull ten weeks after injection with MCF-7 cells stably expressing miR-373, whereas metastasis did not occur following the injection of control MCF-7 cells (Figure 2E). However, TXNIP co-expression in MCF-7 cells stably expressing miR-373, but not TXNIP-3′UTR, blocked miR-373-induced lung metastasis (Figure 2E). Histological analysis showed that miR-373 over-expression led to more massive metastases in the lungs of mice, and TXNIP, but not TXNIP-3′UTR, inhibited miR-373-induced metastasis in the lungs of the mice (Figure 2F). Taken together, these results indicate that miR-373 promotes metastasis mainly by inhibiting TXNIP.

### MiR-373 stimulates EMT and metastasis via TXNIP-dependent ROS reduction

**Figure 3:**
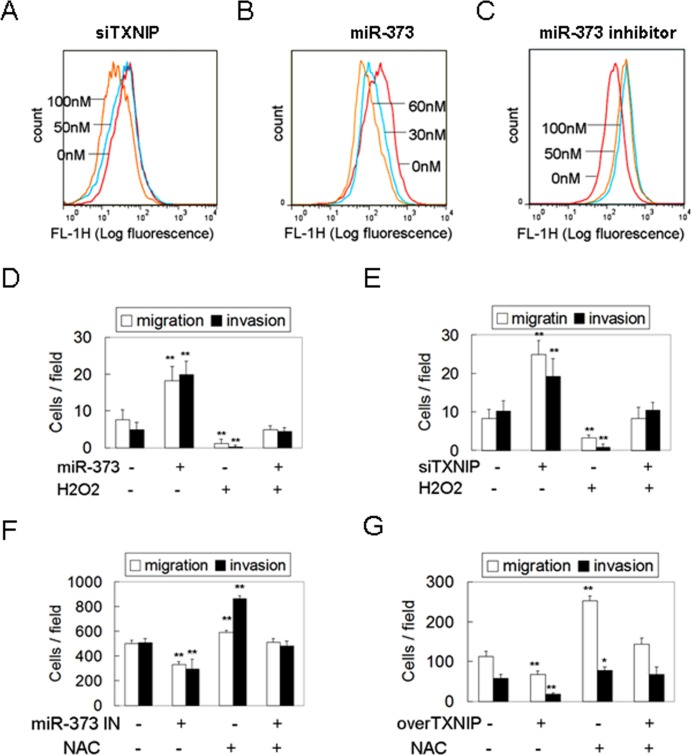
MiR-373 promotes migration and invasion via TXNIP-dependent ROS reduction **A.**, **C.** MCF-7 cells were treated with anti-TXNIP siRNAs **A.** or miR-373 precursor **C.** at the indicated concentration. The intracellular ROS levels were determined by DCFH-DA assay. **B.** MDA-MB-231 cells were treated with miR-373 inhibitor at the indicated concentration. The intracellular ROS levels were determined by DCFH-DA assay. **D.**, **E.** MCF-7 cells were transfected with 30 nM miR-373 precursor **D.** or 50 nM anti-TXNIP siRNAs **E.** following 1 μM H_2_O_2_ treatment (non-toxicity) for 12 h, and then the migration and invasion were analyzed (*n* = 3). **F.**, **G.** MDA-MB-231 cells were transfected with 50 nM miR-373 IN **F.** or TXNIP plasmid **G.** following 2 mM NAC treatment (non-toxicity) for 24 h, and then the migration and invasion were analyzed (*n* = 3).

TXNIP, an endogenous inhibitor of thioredoxin (Trx), binds to the redox-specific active cysteine residues of Trx, thereby negatively regulating Trx activity, rather than changing the Trx level [12]. Trx binds ROS and scavenges ROS before they can harm cells, thus protecting cells against oxidative stress [13]. This suggests that decreased expression of TXNIP releases the inhibition of TXNIP on Trx to decrease intracellular ROS and oxidative stress [14]. Therefore, we asked whether down-regulation of TXNIP by miR-373 decreases the intracellular ROS level to stimulate migration and invasion. As shown in Figure 3A, silencing of TXNIP decreased the intracellular ROS level in a dose-dependent manner. MiR-373 over-expression also resulted in the decrease of intracellular ROS levels (Figure 3B), similar to the effects of TXNIP silencing, whereas miR-373 inhibition had the opposite effect (Figure 3C). Moreover, the enhancement of migration and invasion induced by miR-373 over-expression or TXNIP knockdown was abolished by the non-toxic oxidant, H_2_O_2_ (Figure 3D, 3E, Supplementary Figure S2A), whereas the inhibition of migration and invasion induced by the miR-373 inhibitor or TXNIP over-expression was rescued by the non-toxic antioxidant NAC (Figure 3F, 3G, Supplementary Figure S2B). These results indicate that miR-373 induces migration and invasion primarily via TXNIP-dependent ROS reduction.

### MiR-373 up-regulates HIF1α and TWIST via the TXNIP pathway

**Figure 4:**
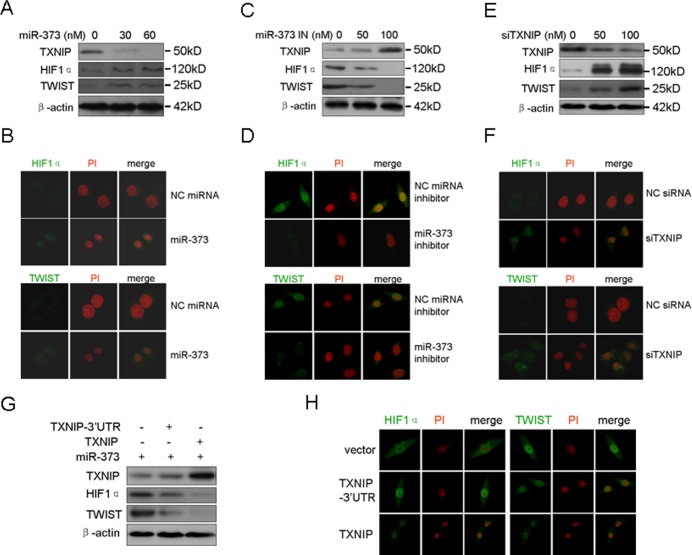
MiR-373 up-regulates and activates HIF1α and TWIST via the TXNIP pathway **A.**, **B.** MCF-7 cells were treated with miR-373 precursor at the indicated concentration, and Western blotting **A.** was then performed as indicated and the protein subcellular location **B.** was analyzed by immunofluorescence assay. HIF1α and TWIST proteins were immunostained (green), and the nuclei were stained with propidium iodide (PI) (red). **C.**, **D.** MDA-MB-231 cells were treated with miR-373 inhibitor (miR-373 IN) at the indicated concentration, and Western blotting **C.** and immunofluorescence **D.** assay were then performed. **E.**, **F.** MCF-7 cells were treated with anti-TXNIP siRNAs at the indicated concentration, and Western blotting **E.** and immunofluorescence **F.** assay were then performed. **G.**, **H.** MDA-MB-231 cells were co-transfected with the indicated plasmids together with the miR-373 precursor, and then Western blotting **G.** and immunofluorescence **H.** assay were performed.

HIF1α and TWIST are two important factors in the induction of tumor EMT and metastasis [15]. Mutant phenotypes of TWIST- and HIF1α-null mice exhibit similarities, including cancer progression and metastasis [15]. Additionally, HIF1α directly increases the expression of TWIST through the HIF1 response element located in the TWIST proximal promoter [15]. Here, we also demonstrated that HIF1α over-expression increased the TWIST level in a dose-dependent manner, whereas silencing of HIF1α down-regulated the TWIST level (Supplementary Figure S3). Early studies showed that ROS are essential for the stability of HIF1α by inactivating PHD2, indicating that elevated ROS increases the HIF1α level by stabilizing HIF1α [16, 17]. Here, we determined whether miR-373 up-regulates the EMT mediators HIF1α and TWIST via the TXNIP pathway because miR-373 down-regulates the intracellular ROS level through TXNIP. As shown in Figure 4A and 4B, miR-373 increased the TWIST and HIF1α level in a dose-dependent manner and induced their nuclear translocation. By contrast, the inhibition of miR-373 resulted in the opposite effect on TWIST and HIF1α (Figure 4C, 4D). The silencing of TXNIP exhibited the expected results on TWIST and HIF1α, similar to miR-373 over-expression (Figure 4E, 4F). Consistently, over-expression of TXNIP, but not TXNIP-3′UTR, abrogated the miR-373-induced up-regulation and nuclear translocation of TWIST and HIF1α (Figure 4G, 4H). Taken together, these results indicate that miR-373 increase the TWIST and HIF1α level and activity by down-regulating TXNIP.

### TWIST induces miR-373 expression

**Figure 5:**
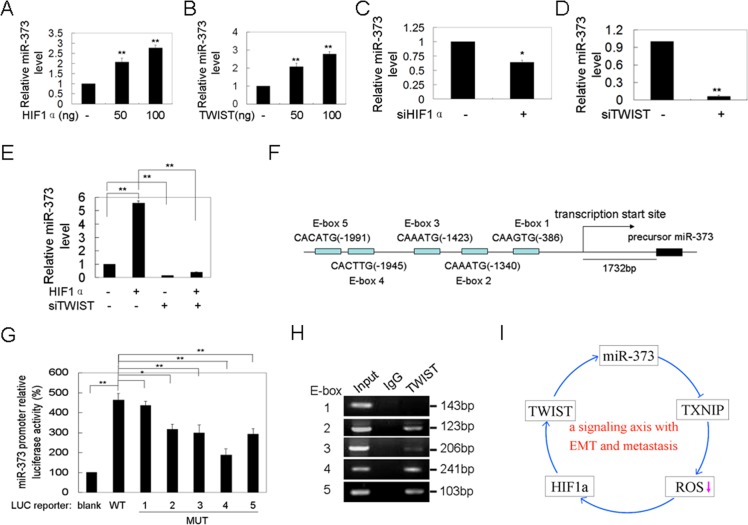
Twist up-regulates miR-373 expression by binding to the promoter of the miR-371-373 gene cluster **A.**, **B.** MCF-7 cells were transfected with HIF1α **A.** or TWIST **B.** plasmid at the indicated concentration for 48 h, and the miR-373 level was then analyzed by qRT-PCR (*n* = 3). **C.**, **D.** MDA-MB-231 cells were transfected with anti-HIF1α **C.** or anti-TWIST **D.** siRNAs at the indicated concentration for 48 h, and the miR-373 level was then analyzed by qRT-PCR (*n* = 3). **E.** MDA-MB-231 cells were co-transfected with HIF1α plasmid and anti-TWIST siRNAs, and then the miR-373 level was analyzed by qRT-PCR (H) (*n* = 3). **F.** A schematic representation of five TWIST binding sites in the promoter of the miR-371-373 gene cluster. **G.** The indicated reporter plasmids were co-transfected with TWIST plasmid into MCF-7 cells, and the luciferase activity was then analyzed (*n* = 3). **H.** The ChIP assay for the TWIST binding sites in the promoter of the miR-371-373 gene cluster. Input, 10% of total input lysates. **I.** A schematic overview of the signaling axis of miR-373 with EMT and metastasis.

A previous study showed that miR-372 and 373 are up-regulated in response to hypoxia. The HIF1α protein level was increased via ROS-dependent HIF1α stabilization under hypoxia conditions. Therefore, we determined whether miR-373-induced TWIST and HIF1α up-regulation and activation increases miR-373 expression. We demonstrate that HIF1α or TWIST over-expression increases miR-373 expression (Figure 5A, 5B), whereas silencing of HIF1α or TWIST reduces the miR-373 level (Figure 5C, 5D). We asked whether HIF1α increases miR-373 expression via TWIST. As shown in Figure 5E, TWIST knockdown attenuated the HIF1α over-expression-induced up-regulation of miR-373, suggesting that TWIST plays a key role in the HIF1α-induced up-regulation of miR-373 expression because a previous study showed that HIF1α directly induces TWIST expression by binding to the promoter of TWIST [15].

Furthermore, the miR-371-373 promoter region was analyzed using the TESS program [18]. Five binding sites (E-box) for TWIST were found in the region approximately 2.0 kb upstream from the transcriptional start site of the miR-371-373 cluster (Figure 5F) where the binding site for HIF1α is not present. Indeed, co-transfection of TWIST resulted in a 4.6-fold increase in miR-371-373 promoter activity (Figure 5G), which was significantly attenuated by individual mutation of the E-box 2, 3, 4, and 5 site, but not E-box 1 (Figure 5G, Supplementary Figure S4). The binding of TWIST to E-box 2, 3, 4, and 5, but not E-box 1, was determined using ChIP assay (Figure 5H). These results suggest that TWIST is a transcription factor that directly binds to the miR-371-373 promoter. Taken together, miR-373-activated TWIST up-regulated miR-373 expression by binding to the promoter of the miR-371-373 cluster. Furthermore, miR-373 induced EMT and metastasis through the miR-373-TXNIP- HIF1α-TWIST signaling axis (Figure 5I).

### The activation of the miR-373-TXNIP-HIF1α-TWIST signaling axis is associated with worse outcomes in patients with breast cancers

**Figure 6:**
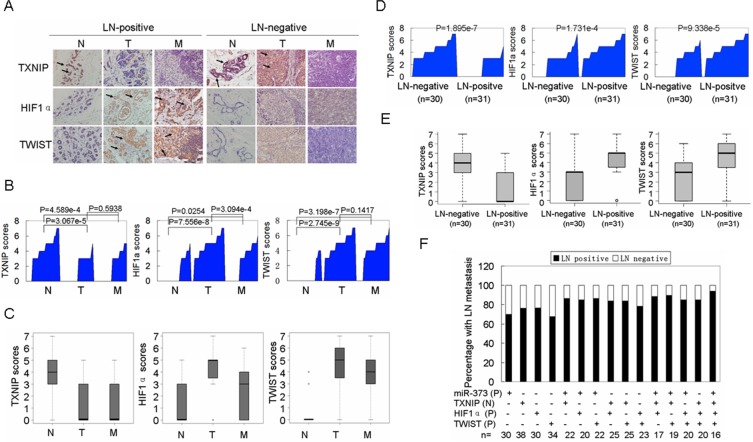
Activation of the miR-373-TXNIP-HIF1α-TWIST signaling axis is associated with worse outcomes in breast cancer patients **A.** Representative IHC images of TXNIP, HIF1α and TWIST expression in adjacent non-cancerous breast tissues (N), primary cancers (T), and lymph node (LN) metastases (M). Images were captured at 400× magnification. **B.** TXNIP, HIF1α and TWIST expression are plotted using their IHC scores in adjacent non-cancerous samples, primary cancers and lymph node metastases in the LN-positive group (*n* = 31). **C.** TXNIP, HIF1α and TWIST expression scores are shown as a box plot with the horizontal lines representing the median. The bottom and top of the boxes represent the 25^th^ and 75^th^ percentiles, respectively, and the vertical bars represent the range of data (*n* = 31). **D.** TXNIP, HIF1α and TWIST levels were plotted for LN-negative and positive primary breast cancers. **E.** TXNIP, HIF1α and TWIST expression scores of LN-negative and positive primary breast cancers are shown in box plots. **F.** The percentage of lymph node metastases was analyzed in patients with miR-373 positive (P), TXNIP negative (N), HIF1α positive (P), and TWIST positive (P) primary cancers. For miR-373, a value above the mean indicates positive (P). For TXNIP, IHC scores (0, +) are interpreted as negative (N). For HIF1α and TWIST, IHC scores (++, +++) are considered positive (P).

Finally, the miR-373-TXNIP-HIF1α-TWIST signaling axis was evaluated for its clinical relevance. As shown in Figure 6A and Supplementary Table S1, the levels of miR-373, TXNIP, HIF1α and TWIST were significantly associated with the TNM stage and lymph node metastasis, but not with other tested clinical characteristics in patients with breast cancer. Moreover, TXNIP was reduced, whereas miR-373, HIF1α and TWIST were increased in primary breast cancers and lymph node metastases compared to the adjacent normal breast tissues (Figure 6B, 6C, Supplementary Figure S5), and these alterations in miR-373, TXNIP, HIF1α and TWIST were also observed in patients with LN-positive primary breast cancers compared to LN-negative primary cancers (Figure 6D, 6E; Supplementary Figure S5, S6). Furthermore, as shown in Supplementary Table S2, negative correlations were observed between miR-373 and TXNIP, TXNIP and HIF1α, and TXNIP and TWIST, whereas positive correlations were observed between miR-373 and HIF1α, miR-373 and TWIST, and HIF1α and TWIST. Importantly, the increase of miR-373, HIF1α or TWIST, or the reduction of TXNIP can be used to predict the risk of metastasis in patients with breast cancer, and the highest percentage of lymph node metastases was observed in patients with a combination of the four factors described above (Figure 6F).

## DISCUSSION

In this study, a novel mechanism by which miR-373 promotes EMT and metastasis through the miR-373-TXNIP-HIF1α-TWIST signaling axis was elucidated in breast cancer. Although miR-373 is up-regulated in breast, testicular germ-cell, colon cancer and fibrosarcoma, and miR-373, as an oncogene, promotes migration and invasion [9, 10, 19, 20], the mechanism by which miR-373 promotes metastasis is poorly understood. As shown by Huang at al., miR-373 promotes migration and invasion partly by targeting CD44 in breast cancer. Here, we further demonstrate that miR-373 stimulates EMT to induce metastasis in breast cancer [10]. MiR-373 up-regulates and activates two important EMT inducers, HIF1α and TWIST, by directly inhibiting TXNIP to drive cancer cell EMT. In turn, TWIST increases miR-373 expression by binding to the promoter of the miR-371-373 gene cluster.

TXNIP is frequently down-regulated in various cancers. However, genetic alternations of TXNIP, such as deletion, translocation or somatic mutation, are not detected in these cancers [21]. TXNIP was first identified as a metastasis suppressor due to the low expression of TXNIP in metastatic melanoma cells caused by Chr6 deletion [22]. TXNIP itself is not located on Chr6; however, relevant regulators of this protein originate from Chr6, such as CRSP3 [22]. Here, we found a novel regulatory mechanism by which TXNIP expression is inhibited by microRNAs, such as miR-373, at the post-transcriptional level in metastatic breast cancers.

TXNIP, as an endogenous inhibitor of Trx, inhibits the antioxidative function of Trx, which reduces intracellular ROS level through its free thiol at two cysteine residues [23]. In this study, we found that miR-373 over-expression and silencing of TXNIP decreased the intracellular ROS level and up-regulated and activated HIF1α and TWIST. The non-toxic oxidant H_2_O_2_ attenuated miR-373 over-expression, and TXNIP knockdown-induced migration and invasion. The non-toxic anti-oxidant NAC reversed the migration and invasion inhibition induced by miR-373 inhibition and TXNIP over-expression. These findings indicate that miR-373 promotes migration and invasion via TXNIP-dependent ROS reduction, suggesting that ROS reduction in cancer cells promotes migration and invasion. In the “canonical” view, an over-accumulation of ROS results in cell damage, aging, cell senescene and apoptosis, whereas mild elevations in ROS act as a signaling intermediate, promoting carcinogenesis, malignant progression, invasion and metastasis [24]. Recently, new evidence showed that cancer stem cells (CSCs) in human and murine breast tumors contain a lower concentration of ROS than corresponding non-tumorigenic cells [25]. Metastatic cancer cells or cancer cells that have undergone the EMT and CSCs share several common characteristics and properties [26]. Dong et al. demonstrated that loss of FBP1 by Snail-induced repression decreases the intracellular ROS level to increase the CSC-like property and EMT of cancer cells in basal-like breast cancer [27]. Taken together, we propose that the invasive and metastatic ability of cancer cells may be induced by the reduction of ROS, which can be achieved by *in vivo* intrinsic gene dysregulation, such as the silencing of TXNIP as demonstrated here or of FBP1 as reported recently [27] or by *in vitro* extrinsic factors, such as antioxidant NAC treatment.

HIF1α is an important micro-environmental factor that induces the expression of certain regulators, such as TWIST, Snail, SIP1, Zeb1 and E47, to promote EMT and metastasis [15, 28, 29]. HIF1α directly induces the expression of TWIST through the HIF1 response element located in the TWIST proximal promoter [15]. Here, we show that miR-373 elevates and activates HIF1α and TWIST by directly inhibiting TXNIP. TXNIP co-expression, but not the TXNIP-3′UTR, blocks the increased migration and invasion and up-regulation and activation of HIF1α and TWIST induced by miR-373. HIF1α and TWIST are two important EMT inducers in cancer cells, and TWIST is an important downstream effector of HIF1α. Therefore, the miR-373-induced HIF1α-TWIST signaling axis stimulates cancer cell EMT and metastasis.

A previous study showed that miR-372 and 373 are up-regulated in response to hypoxia via HIF1a and TWIST [20]. However, in this report, the TWIST binding sites were in the promoter of the predicted transcript of the miR-371-373 cluster, derived from its mouse homolog, miR-290-295 [20]. Because of the poor conservation of genome organization between the two homologs, the full-length primary transcript of the miR-371-373 cluster was determined by 5′RACE and 3′ RACE by the Qu LH group [30]. Our BLAST assay demonstrated that the full-length primary transcript of the miR-371-373 cluster is different from the predicted transcript. Therefore, in this study, the promoter of the full-length primary transcript of the miR-371-373 cluster was re-analyzed. We show that TWIST directly induces the expression of the miR-371-373 cluster by binding to four novel binding sites in the promoter of the miR-371-373 cluster, suggesting that the HIF1a-TWIST signaling axis in turn induces miR-373 expression. In clinical breast cancer tissues, we further demonstrate that miR-373 is positively associated with HIF1α and TWIST, and negatively associated with TXNIP. Activation of the miR-373-TXNIP-HIF1α-TWIST signaling axis is associated with worse outcomes in the patients with breast cancers, suggesting that the signaling axis is a potential biomarker or therapeutic target for breast cancer.

In conclusion, our findings indicate that miR-373 drives cancer cell EMT. A novel mechanism by which miR-373 promotes EMT and metastasis though the miR-373-TXNIP-ROS-HIF1α-TWIST signaling axis is elucidated in breast cancer. Activation of the signaling axis may be a potential biomarker for metastasis and a therapeutic target. In addition, intracellular ROS reduction induced by miR-373 up-regulation or TXNIP silencing promotes the EMT phenotype, migration and invasion by activating the HIF1α and TWIST pathway.

## MATERIALS AND METHODS

### Cell culture and tissue samples

MDA-MB-231, MDA-MB-435s and MCF-7 breast cancer cell lines were obtained from the American Type Culture Collection and cultured under standard conditions. Clinical samples, including tissues from primary breast cancers, lymph node metastasis, and adjacent, non-tumor sites, were obtained at the time of surgery from 61 previously untreated breast cancer patients at the Third Affiliated Hospital of Guangzhou Medicine University and the Nanfang Hospital of the Nanfang University of Medicine from March 2010 to May 2012. Pathological diagnosis, as well as ER, PR, Her2, and Ki-67 status was verified by two independent pathologists. Informed consent was obtained from each patient, and the Internal Review and Ethics Boards at the Third Affiliated Hospital of Guangzhou Medicine University and the Nanfang Hospital of the Nanfang University of Medicine approved collection of the tissue specimens.

### Antibodies

Antibodies against TXNIP were obtained from Invitrogen (Cat. 40-4600, for WB) and Sigma-Aldrich (Cat. HPA031085, for IHC). Antibodies for HIF1α were from ProteinTech (Cat. 20960-1-AP, for WB and IF) and Epitomics (Cat. 2015-1, for IHC). Anti-Twist1 antibodies were from Abcam (Cat. Ab50887, for IHC) and Sigma-Aldrich (Cat. SAB1406563, for WB and IF). The EMT antibody sampler kit was from Cell Signaling (Cat. 9782, for WB).

### Oligonucleotide transfection

The miRNA-373 precursor or inhibitor (Ambion) and siRNA duplexes targeted to TXNIP, HIF1α or TWIST (GenePharma) were transfected with RNAiMAX (Invitrogen). The reporter plasmids, TXNIP, TXNIP-3′UTR vector and DNA-RNA (miRNA mimics or inhibitor) mix were transfected using Lipofectamine 2000 (Invitrogen). Forty-eight hours after transfection, unless otherwise stated, cells were harvested for analysis using the luciferase reporter assay, Western blotting, ROS measurements, immunofluorescence detection, and migration and invasion assays.

### Constructs

The TXNIP 3′UTR sequence and a region approximately 2.0 kb upstream from the transcriptional start site of the miR-371-373 cluster were cloned into the psiCHKECK-2 or pGL3 luciferase vectors (Promega), respectively. The TXNIP cDNA sequence and TXNIP-3′UTR full sequence containing the TXNIP 3′UTR region were cloned into the pcDNA3.1/6His vector (Invitrogen). The HIF1α and TWIST cDNA were cloned into pcDNA3.1 plasmid (Invitrogen). The mutants of the miR-371-373 promoter reporter constructs were generated using a QuikChange Site-Directed Mutagenesis Kit (Stratagene).

### Western blotting

The proteins were detected using antibodies according to the method previously described [31]. Briefly, total proteins were separated by 10% SDS-PAGE and then electroblotted onto a polyvinylidene fluoride (PVDF) membrane. The membranes were incubated with the indicated primary antibodies at 4°C overnight, followed by incubation with the corresponding secondary antibodies for 1 h. The antibody-bound proteins were further detected using a chemiluminescence assay (Pierce, USA), followed by exposure to autoradiographic film in a darkroom.

### Construction of stable expression cell lines

To establish the luciferase-labeled cell lines, the lentivirus pLeno-RFP-LUC that expresses red fluorescence protein (RFP) and luciferase was purchased from Invabio (Shanghai, China). MCF-7 cells were transduced with the lentivirus, pLeno-RFP-Luc. The Luc-labeled MCF-7-Luc cells were sorted by flow cytometry and selected using puromycin.

To establish the miR-373 over-expression cell lines, the lentivirus pLV3-373-GFP that expresses miR-373 and green fluorescence protein (GFP) was purchased from GenePharma (Shanghai, China). The established MCF-7-Luc cells were further transduced with the lentivirus, pLV3-373-GFP or pLV3-NC-GFP. The cell lines MCF-7-Luc-373 and MCF-7-Luc-VEC stably expressing miR-373 and a blank vector were constructed, respectively. The efficiency of miR-373 over-expression was determined by quantitative PCR.

To establish the TXNIP or TXNIP-3′UTR over-expression cell lines, lentivirus constructs, pLV4-TXNIP-GFP that expresses TXNIP and GFP and pLV4-TXNIP3′UTR-GFP that contains the TXNIP-3′UTR and expresses GFP, were purchased from GenePharma. The established MCF-7-Luc-373 cells were further transduced with the lentiviruses, pLV4-TXNIP-GFP or pLV4-TXNIP3′UTR-GFP. The MCF-7-Luc-373-TXNIP and MCF-7-Luc-373-TXNIP3′UTR cell lines stably expressing miR-373/TXNIP and miR-373/TXNIP-3′UTR were constructed, respectively.

### Animal studies

Female NOD-SCID mice (5-6 weeks old) were purchased from Charles River Laboratories in China (Beijing) and bred and maintained under defined conditions at the Animal Experiment Center of College of Medicine (SPF grade), Jinan University. *In vivo* tail vein metastatic assays were performed as previously described with minor modifications [10, 15]. In brief, 4×10^6^ Luc-labeled cells stably expressing miR-373, miR-373/TXNIP-3′UTR, miR-373/TXNIP, and their NCs, MCF-7-373, MCF-7-373-TXNIP3′UTR, MCF-7-373-TXNIP and MCF-7-NC, were injected into the tail veins of female NOD-SCID mice. The metastatic foci in the lungs were visualized using the IVIS 200 Imaging System (Xenogen) ten weeks after implantation. These mice were then euthanized before dissection, and the lung tissues were subsequently fixed in 10% formalin and prepared for standard histological examination. Animal experiments were approved by the Laboratory Animal Ethics Committee of Jinan University and conformed to the US Department of Health and Human Services Guide for the Care and Use of Laboratory Animals.

### Measurement of ROS

The intracellular ROS levels were determined by measuring the oxidative conversion of cell permeable 2′,7′-dichlorofluorescein diacetate (DCFH-DA) to fluorescent dichlorofluorescein (DCF) using a fluorospectrophotometer as previously described [32].

### Migration and invasion assays

*In vitro* migration and invasion assays were performed using Transwell chambers as previously described [11]. Briefly, MCF-7 or MDA-MB-231 cells were transfected with the indicated concentrations of the indicated siRNAs or plasmids, or co-transfected with the indicated siRNA or miRNAs and plasmid. These cells were then detached and resuspended in serum-free medium after 48 h of transfection. The cell suspensions were added in the upper Transwell chambers for the migration assay (8.0 μM pore size, BD), or the upper Transwell chambers coated with Matrigel for the invasion assay. Cells on the undersurface were stained with 5% crystal violet. Images were captured and the number of migratory or invasive cells was counted under a microscope.

### Real-time qRT-PCR for miRNA

Total RNA was extracted using the Trizol total RNA isolation reagent (Invitrogen). Detection of the mature form of miR-373 was performed using a Hairpin-it-miRNAs qPCR quantitation kit and qRT-PCR primer sets according to the manufacturer's instructions (GenePharma, Shanghai, China). Briefly, microRNAs were reverse transcribed using a stem-loop RT primer. Then, quantitative PCR was performed (*n* = 3). The U6 small nuclear RNA was used as an internal control.

### Luciferase assay

The TXNIP-3′UTR luciferase reporter gene constructs and their mutation constructs were transfected into MCF-7-373 cells stably expressing miR-373 or co-transfected with the miR-373 inhibitor into MDA-MB-231 cells in 24-well plates. Firefly luciferase activity was used to normalize the Renilla luciferase activity for each transfected well (*n* = 3). The miR-371-373 firefly luciferase reporter gene constructs or their mutation constructs were co-transfected with the TWIST plasmid into MCF-7 cells for 48 h. The pRL-TK Renilla luciferase reporter vector was used as an internal control reporter vector (Promega). Luciferase activity was measured using the dual-luciferase reporter assay system (Promega). Renilla luciferase activity was used to normalize the firefly luciferase activity (*n* = 3).

### Immunofluorescence staining

Cells were transfected with the miR-373 precursor, miR-373 inhibitors, siRNA targeting TXNIP, a TXNIP vector or the TXNIP-3′UTR vector for 24 h. The transfected cells were then detached and re-cultured on glass cover slips for 24 h. Cells were fixed with 4% paraformaldehyde and permeabilized with 0.1% Triton X-100. These cells were incubated with an anti-HIF1α or TWIST antibody and then reacted with a corresponding Alexa Fluor 488-conjugated IgG. Cellular nuclei were stained with propidium iodide (PI). Staining for HIF1α, TWIST and PI was visualized and captured using a confocal laser microscope (Nikon, Japan).

### Immunohistochemistry (IHC) staining

IHC was performed according to a standard LSAB protocol (Dako, Carpinteria, CA) using anti-TXNIP, HIF1α and TWIST antibodies. All staining was assessed by two independent pathologists blinded to the sample origination and subject outcome. The German semi-quantitative scoring system that determines the staining intensity and area of extent was used as previously described with minor modifications [33, 34]. Nuclear HIF1α or TWIST staining was evaluated as previously described based on the observation that the nuclear localization of HIF1α and TWIST represent the biologically active form of these transcription factors [34]. Briefly, each specimen was assigned a score according to the extent of cell staining 0 (≤10%), 2 (11-50%), 3 (51-80%) and 4 ( > 80%) and the staining intensity 0 (no staining), 1 (slight staining), 2 (moderate staining), and 3 (strong staining) [33]. The total scores represent the extent and intensity scores ranging from 0 to 7. Using the total score, specimens were classified into one of four groups as follows: negative expression (−), less than 10% of cells stained positive regardless of intensity; slight expression (+), 3; moderate expression (+ +), 4–5; and strong expression (+ + +), 6–7.

### Statistical analysis

The two-tailed independent Student's t-test and the Mann Whitney U test were used for comparisons between two groups. The correlations were determined using the Spearman's rho correlation test. The data are presented as the mean ± SD, except where stated otherwise. All statistical analyses were performed using SPSS (Version 16.0). Values of *p < 0.05 and **p < 0.01 were considered statistically significant.

## SUPPLEMENTARY MATERIALS FIGURES AND TABLES



## Abbreviations

EMT: epithelial-mesenchymal transition
TXNIP: thioredoxin-interacting protein
Trx: thioredoxin
ROS: reactive oxygen species
CSCs: cancer stem cells
