# Supplementary material for: MiR-373 drives the epithelial-to-mesenchymal transition and metastasis via the miR-373-TXNIP-HIF1α-TWIST signaling axis in breast cancer
Source: Oncotarget. 2015 Jul 1;6(32):32701–12. doi: 10.18632/oncotarget.4702 (PMC4741723; doi:10.18632/oncotarget.4702)
Supplement: Supplementary file 1 [file oncotarget-06-32701-s001.pdf]

MiR-373 drives the epithelial-to-mesenchymal transition and metastasis via the miR-373-TXNIP-HIF1α-TWIST signaling axis in breast cancer

Supplementary Materials

Supplementary Figures

Figure S1

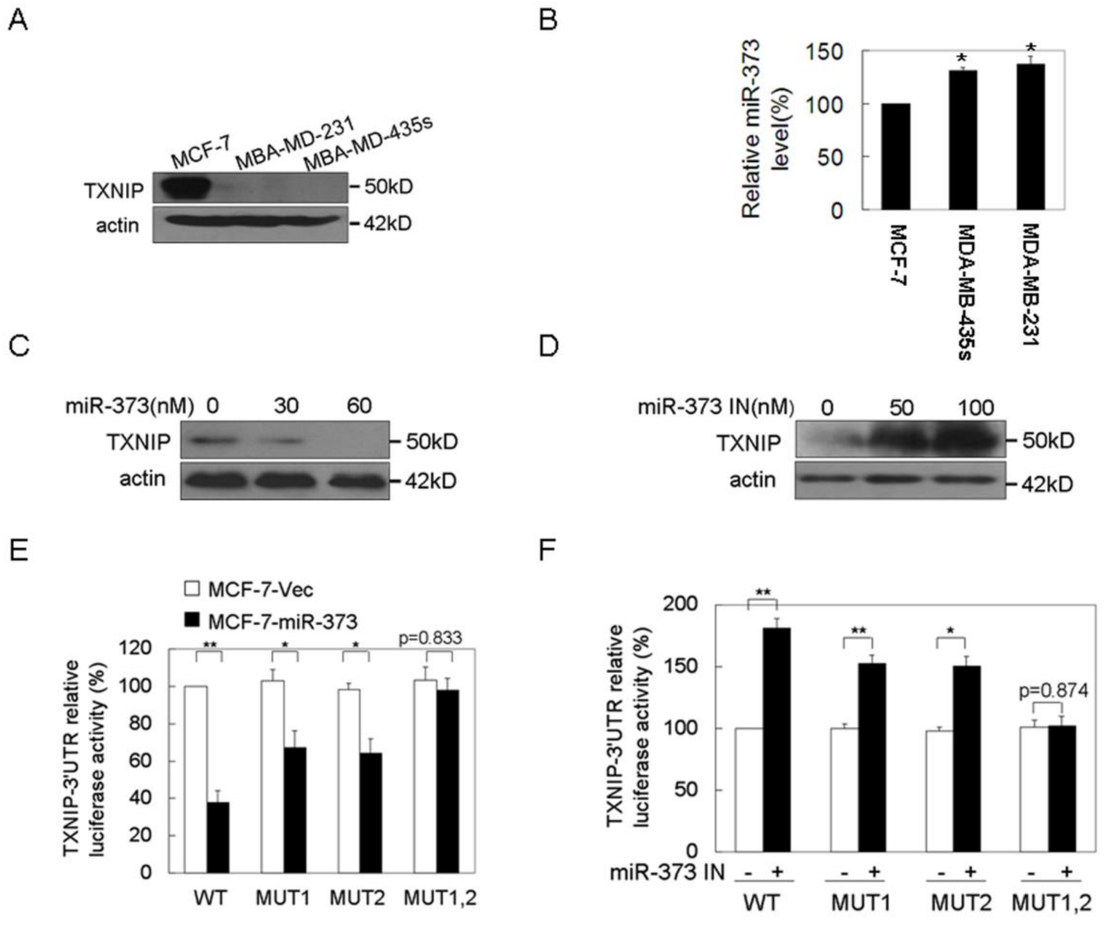

Figure S1. TXNIP is a direct target of miR-373. (A, B) TXNIP and miR-373 levels were determined in the indicated breast cancer cell lines with varying metastatic

abilities by western blotting and qRT-PCR, respectively. (C) MCF-7 cells were treated with the indicated concentration of miR-373 precursor, TXNIP levels were detected by western blotting. (D) MBA-MD-231 cells were treated with the indicated concentration of miR-373 inhibitor (miR-373 IN), TXNIP levels were detected by western blotting. (E) The MCF-7-373 cells stably expressing miR-373 or blank vector were transfected with the indicated reporter plasmids. Luciferase activity was measured 48 h after transfection using the dual-luciferase reporter assay system. (F) MDA-MB-231 cells were co-transfected with 50 nM miR-373 IN and the indicated reporter plasmids. Luciferase activity was measured as described above in b (n=3).

Figure S2

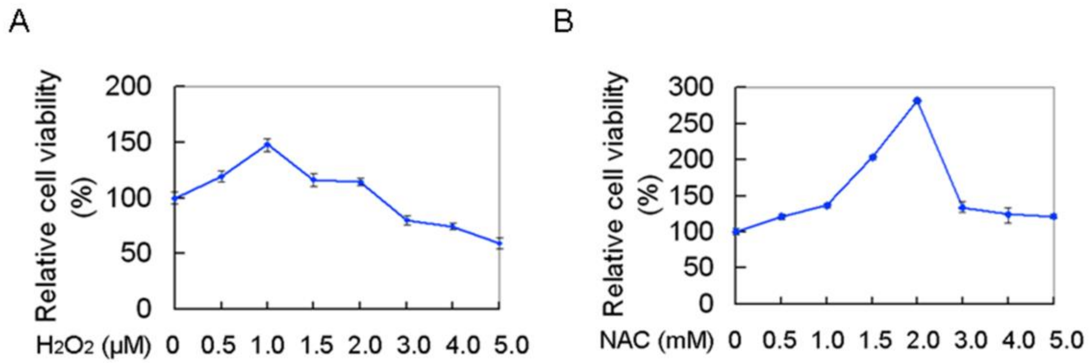

Figure S2. Cell toxicity was not induced by low-dose oxidant H_2O_2 and antioxidant NAC, respectively. MCF-7 cells were treated with oxidant H_2O_2 (A) and antioxidant NAC (B) at the indicated concentrations, and then the cell viability was measured by WST-1 assay.

Figure S3

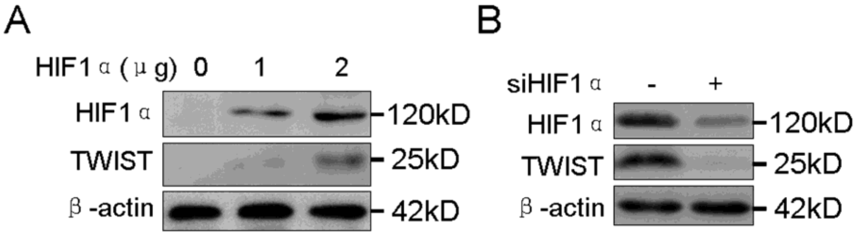

Figure S3. TWIST level was up-regulated by HIF1α. (A) MCF-7 cells were transfected with HIF1α plasmid at the indicated amount for 48h, HIF1α and TWIST levels were determined by western blotting. (B) MDA-MB-231 cells were transfected with anti-HIF1α siRNAs for 48h, HIF1α and TWIST levels were determined by western blotting.

Figure S4

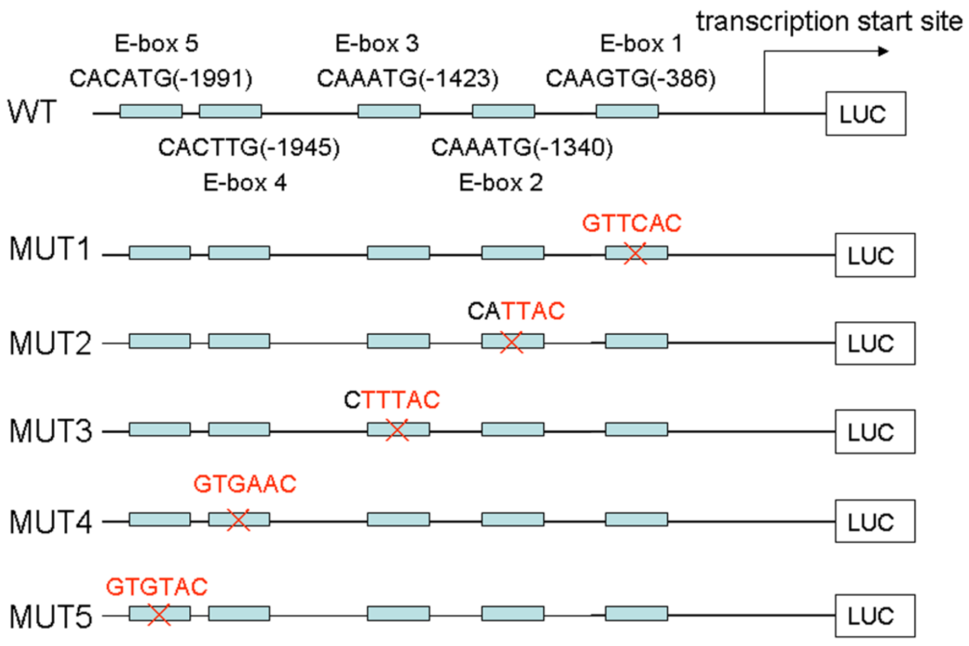

Figure S4. A schematic representation of five binding sites in the promoter region of the miR-371-373 gene cluster and reporter constructs used in Figure 5F. The constructs were either wild-type or contained 5 mutations of the E-box. The base sequence labeled in red indicates the mutated base in each construct.

Figure S5

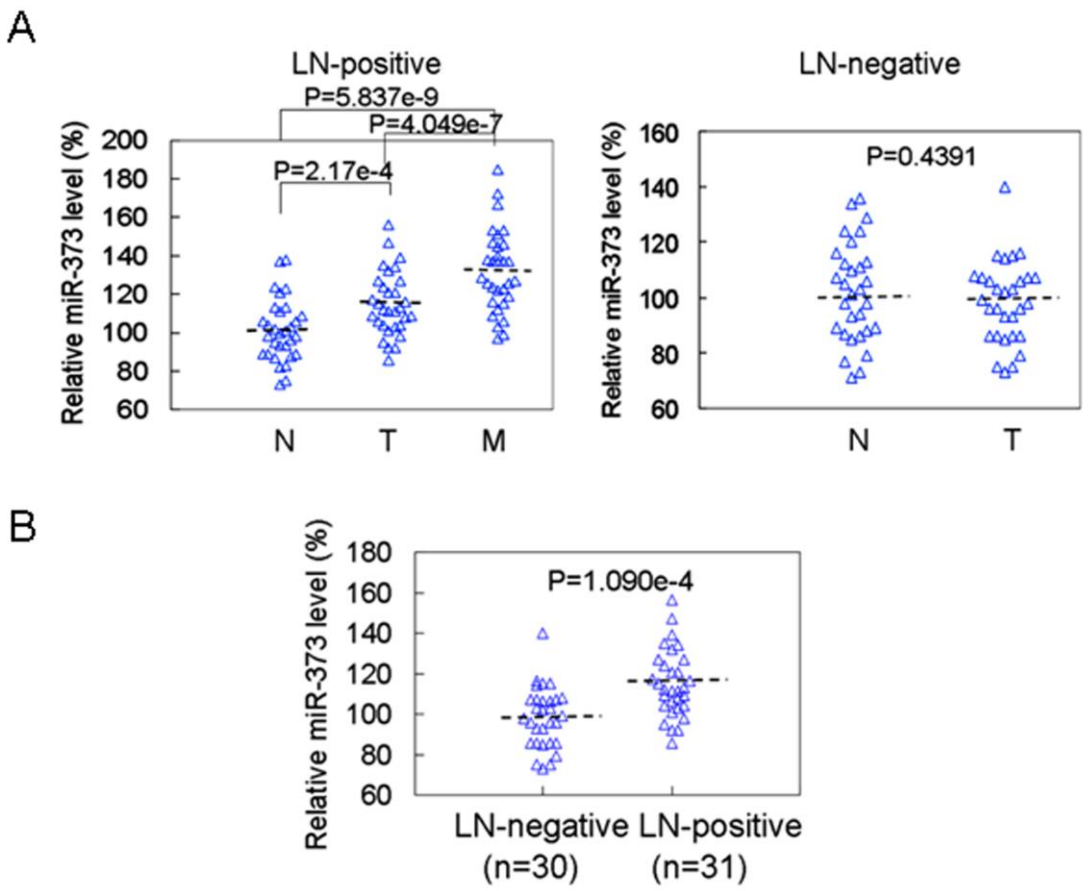

Figure S5. MiR-373 level was up-regulated in breast cancers and lymph node metastases compared to adjacent non-tumor breast tissues, and up-regulated in LN-positive breast cancers compared to LN-negative breast cancers. (A) The miR-373 levels in adjacent non-tumor breast tissues (N), breast cancer tissues (T), and lymph node metastases (M) were compared among 31 LN-positive cases. The dotted lines

represent the mean value for each sample group. (B) The levels of miR-373 expression were compared between 30 LN-negative and 31 LN-positive primary breast cancers.

Figure S6

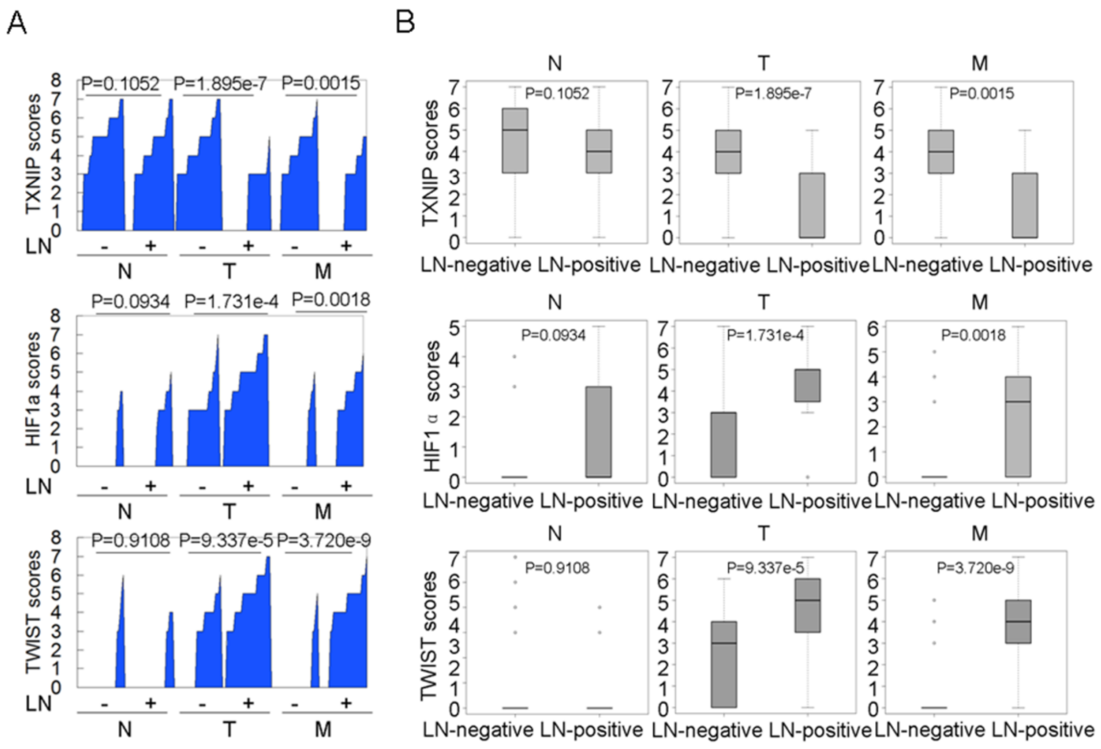

Figure S6. HIF1α and Twist expression were markedly increased, whereas TXNIP expression was reduced in primary cancers (T) and lymph node metastases (M) between LN-positive (n=31) and LN-negative (n=30) groups, whereas they were not different in adjacent normal tissues (N) between the LN-positive and LN-negative groups. TXNIP, HIF1α and TWIST expression scores between the LN-positive and LN-negative in adjacent normal tissues, primary cancers and lymph node metastases (or lymph node tissues) are shown as a plot (A) or a box plot (B) as outlined in

Figures 6D and 6E.

Supplemenatry Tables

Supplemenatry Table S1. Correlation of miR-373, TXNIP, HIF1α and TWIST with clinicopathological status in 61 cases of patients with breast cancer (30 lymph node-negative and 31 lymph node-positive).

	miR-373[a]		P	TXNIP				P	HIF1α				
	(-)	(+)		-	+	++	+++		-	+	++	+++	
Age (years)			0.1134					0.5791					0.3[illegible]
≤50	18	24		11	16	10	5		10	9	15	8	
＞50	7	12		8	3	7	1		3	9	16	1	
Tumor Size (cm)[b]			0.6331					0.8673					0.1[illegible]
T1 (≤2)	5	11		3	6	5	2		2	4	6	4	
T2 (2-5)	14	18		13	9	8	2		6	12	10	4	
T3 (＞5)	6	6		3	4	4	2		5	2	5	1	
TNM stage			0.0098					1.1287E8					0.0[illegible]
Ⅰ	5	3		0	1	5	2		1	4	1	2	
Ⅱ	16	7		3	6	10	4		9	9	4	1	
Ⅲ	4	26		16	12	2	0		3	5	16	6	
Histological Grade			0.1983					0.2463					0.1[illegible]
G1-G2	14	17		8	10	8	5		10	8	8	5	
G3	11	19		11	9	9	1		3	10	13	4	
Lymph Node Metastasis[b]			2.4361 E6					3.6976E8					6.4[illegible]
N0	21	9		2	7	15	6		10	13	5	2	
N1	0	3		2	10	1	0		0	0	1	2	
N2	2	9		6	5	0	0		3	3	4	1	
N3	2	15		9	7	1	0		0	2	11	4	
ER			0.8315					0.9743					0.7[illegible]
(-)	6	13		5	8	4	2		3	8	6	2	
(+)	19	23		14	11	13	4		10	10	15	7	
PR			0.3173					0.0491					0.9[illegible]
(-)	7	18		11	8	4	2		3	10	10	2	
(+)	18	18		8	11	13	4		10	8	11	7	
HER2			0.4688					0.5499					0.0[illegible]
(-)	18	26		12	15	13	4		11	12	14	7	
(+)	7	10		7	4	4	2		2	6	7	2	
Ki-67			0.5022					0.8988					0.3[illegible]
(-)	7	6		3	5	5	0		2	2	8	1	
(+)	18	30		16	14	12	6		11	16	13	8	

[a]miR-373 level with more than the median is as (+), miR-373 level with less than the median is as (-).
[b]Tumor Size and Lymph Node Metastasis is staged according to the Seventh Editions of the American Joint Committee on Cancer (AJCC) Cancer Staging Manual and Handbook.

Supplemenatry Table S2. Correlations of the IHC expression gradient of miR-373, TXNIP, HIF1α and TWIST in primary samples of 61 breast cancer cases.

a. Correlations of the expression gradient of miR-373 with TXNIP, HIF1α and TWIST in primary samples of 61 breast cancer cases.

	TXNIP				P	HIF-1α				P	TWIST				P
	(-)	(+)	(++)	(+++)		(-)	(+)	(++)	(+++)		(-)	(+)	(++)	(+++)	
miR-373					0.0032(r= -0.4326)					0.0003(r= 0.5689)					0.0183(r= 0.3445)
(-)	3	6	13	3		10	10	4	1		12	3	8	2	
(+)	16	13	4	7		3	8	17	8		5	7	17	7	

b. Correlations of the IHC expression gradient of TXNIP with HIF1α and TWIST in primary samples of 61 breast cancer cases.

	HIF-1α				P	TWIST				P
	(-)	(+)	(++)	(+++)		(-)	(+)	(++)	(+++)	
TXNIP										0.0022(r= -0.385)
0.0250(r= -0.2867)										
(-)	1	5	10	3		3	3	9	4	
(+)	5	2	7	5		5	2	9	3	
(++)	5	7	4	1		7	1	7	2	
(+++)	2	4	0	0		2	4	0	0	

c. Correlation of the IHC expression gradient of HIF1α with TWIST in primary samples of 61 breast cancer cases.

	HIF-1α				P
	(-)	(+)	(++)	(+++)	
TWIST					0.0011(r=0.4089)
(-)	8	2	2	1	
(+)	5	6	5	2	
(++)	1	2	15	3	
(+++)	3	0	3	3	
